# The effects of the “living high–training low” model on key proteins involved in cellular aerobic and anaerobic metabolism in male C57BL/6J mice

**DOI:** 10.14814/phy2.70812

**Published:** 2026-03-24

**Authors:** Pedro P. M. Scariot, Marcelo Papoti, Emanuel E. C. Polisel, Juan B. Orsi, David W. Hill, Fúlvia B. Manchado‐Gobatto, Claudio A. Gobatto

**Affiliations:** ^1^ Laboratory of Applied Sport Physiology University of Campinas—UNICAMP Limeira Sao Paulo Brazil; ^2^ Hypoxia, Sport and Health Team—HSHT Campinas São Paulo Brazil; ^3^ School of Physical Education and Sport of Ribeirão Preto University of São Paulo—USP Ribeirao Preto São Paulo Brazil; ^4^ Applied Physiology Laboratory University of North Texas—UNT Denton Texas USA

**Keywords:** hypoxia, mice, protein expression, training

## Abstract

This study aimed to investigate the effects of the “living high–training low” (LHTL) model, in which mice were housed under hypoxic conditions but trained in normoxia to maintain training quality. We analyzed key proteins involved in cellular metabolism and oxidative stress, including hypoxia‐inducible factor 1α (HIF‐1α), peroxisome proliferator‐activated receptor gamma coactivator 1 alpha (PGC‐1α), and oxidation resistance protein 1 (OXR1). Forty male isogenic C57BL/6J mice were divided into nontrained (N) and trained (T) groups living in normoxic (NOR) or hypoxic (HYP) environments. HYP mice were housed 18 h/day for 8 weeks in a normobaric tent supplied with oxygen‐depleted air (FiO_2_ = 14.5%). Animal handling, including training, was conducted in normoxia (FiO_2_ = 19.5%). Training occurred 5 times/week (40 min/session) at ~80% of individual critical velocity. All mice were euthanized to extract the soleus, white gastrocnemius, and hypothalamus for protein analysis. Compared with training alone, LHTL did not lead to significantly greater HIF‐1α, PGC‐1α, or OXR1 expression across tissues; however, induced behavioral changes—such as reduced spontaneous physical activity and diminished ability to complete training—may indicate that the demands of the LHTL model were not trivial.

## 1 INTRODUCTION

Exposure to hypoxic environments, either at natural altitude or in artificial settings such as chambers or tents, has been used for decades to stimulate adaptations aimed at improving physical performance (Beidleman et al., 2006; Bonne et al., 2015; Bor‐Kucukatay et al., 2014; Chapman et al., 2014; Joanny et al., 2001). Although no single model has been identified as optimal, evidence suggests that the living high–training low (LHTL) model is particularly attractive from a practical standpoint because training occurs under normoxia while hypoxic exposure takes place outside exercise sessions, typically for at least 12 h·day^−1^ (Brugniaux et al., 2006; Girard et al., 2020; Levine & Stray‐Gundersen, 1997; Walsh & Whitham, 2006). Despite the logistical and practical differences among hypoxic models, a key question remains whether these approaches effectively translate into improved performance. Addressing this question may require moving beyond performance outcomes alone and incorporating biomolecular analyses since molecular adaptations ultimately contribute to functional changes. Examining biomolecular responses may help elucidate how cellular signaling pathways are regulated by external stimuli. These stimuli may be applied together, as in living and training at altitude, or separately, as in the LHTL model. Although temporally separated, the LHTL stimuli impose a cumulative dual physiological challenge, which constitutes the core complexity when combining these strategies. This raises a critical question: how do hypoxia and exercise training interact in vivo (synergistically or antagonistically) when the objective is to enhance aerobic fitness?

One possible perspective in this context is that hypoxia and exercise training interact synergistically, as each stimulus is thought to target complementary determinants of aerobic performance, such as oxygen delivery and oxygen utilization (Li et al., 2020). This view is largely based on evidence from isolated pathways, in which hypoxia‐induced improvements in oxygen delivery (e.g., angiogenesis and erythropoiesis) are linked to HIF‐1α signaling (Dewi & Fatchiyah, 2013; Hickey & Simon, 2006; Maxwell & Salnikow, 2004; Pugh & Ratcliffe, 2003; Seagroves et al., 2001; Semenza, 2000, 2011), whereas aerobic training–induced improvements in oxygen utilization are associated with mitochondrial pathways regulated by PGC‐1α (Knutti & Kralli, 2001; Lin et al., 2002; Sandri et al., 2006; Wang et al., 2017; Wu et al., 1999). While this is conceptually coherent, at least three biochemical mechanisms described below point to an alternative perspective in which hypoxia and aerobic training may act antagonistically (conflicting) when the primary outcome of interest is aerobic metabolic adaptation (Mason et al., 2007).

First, activation of HIF‐1α and PGC‐1α may impose competing cellular priorities, with hypoxia favoring a more glycolytic phenotype while aerobic training promotes an oxidative phenotype (Horscroft & Murray, 2014; Taylor & Scholz, 2022; Weidemann & Johnson, 2008). Simultaneous engagement of these pathways may therefore dilute or redirect adaptive signals rather than reinforce them. Second, hypoxia‐induced stabilization of HIF‐1α inhibits mitochondrial aerobic metabolism, potentially through mechanisms involving PDK1‐mediated inhibition of pyruvate dehydrogenase (PDH), which limits substrate entry into the tricarboxylic acid cycle (De Palma et al., 2007; Kim et al., 2006; Kirito et al., 2009; Lindholm & Rundqvist, 2016; Papandreou et al., 2006; Shohet & Garcia, 2007; Slot et al., 2014; Solaini et al., 2010). Third, redox regulation represents an additional layer of potential conflict. Hypoxic exposure is associated with increased reactive oxygen species (ROS) production (Chen et al., 2012; Clanton, 2007; Dosek et al., 2007; Joanny et al., 2001; Magalhaes et al., 2004; Maiti et al., 2006; Mazure et al., 2011; Park & Kehrer, 1991; Radák et al., 1994; Ribon et al., 2016), which can further stabilize HIF‐1α (Bell et al., 2007; Hamanaka & Chandel, 2009; Poyton et al., 2009; Sullivan et al., 2016; Verschoor et al., 2010). Despite the presence of antioxidant defenses that buffer hypoxia‐induced ROS, the concomitant exposure to aerobic training and hypoxia may lead to an amplification of ROS production, potentially establishing a state of oxidative stress. Such a scenario could impair muscle contractile function and recovery, mitochondrial efficiency, thereby attenuating gains in aerobic fitness. Taken together, these mechanisms suggest that the interaction between hypoxia and aerobic training is more complex than often assumed, and much of the proposed synergy or antagonism remains speculative due to limited in vivo evidence combining both stimuli within the same intervention framework. Given the scarcity of animal studies using the LHTL model, it is reasonable and informative to focus on key and classical regulatory proteins. While HIF‐1α and PGC‐1α are established regulators of metabolism, OXR1 remains comparatively underexplored despite emerging evidence linking it to cellular antioxidant defense and DNA protection (Durand et al., 2007; Jaramillo‐Gutierrez et al., 2010; Matsui et al., 2020; Wang et al., 2019; Zhang et al., 2018).

Therefore, the main aim of the present work was to investigate the effects of 8 weeks of aerobic training on mice living in normoxia or hypoxia on the protein expression of HIF‐1α, PGC‐1α and OXR1. We studied mouse skeletal muscles with distinct metabolic profiles: the gastrocnemius, known for its higher proportion of type IIb fibers, which are considerably less oxidative, and the soleus, which predominantly contains type I and IIa fibers (Augusto et al., 2004; Hämäläinen & Pette, 1993; Vechetti Jr. et al., 2019). Considering that some tissues are more sensitive to reductions in oxygen availability (and therefore more susceptible to hypoxia), we also studied the same proteins in the hypothalamus, a region containing neurons highly dependent on aerobic metabolism and involved in energy homeostasis (Bergersen, 2007; Blouet & Schwartz, 2010; Elizondo‐Vega et al., 2015; Hillebrand et al., 2002; Koob & Annau, 1973; Scariot et al., 2022; Todd, 2014). Given that the physiological demands resulting from the combination of hypoxia and aerobic training are far from trivial and may impose energetic and muscular challenges, a secondary objective was to assess induced behavioral changes. Specifically, we evaluated the animals' ability to complete training workouts, even when exercise sessions were performed under normoxic conditions. In addition, we also recorded the spontaneous physical activity (SPA) throughout the intervention, given evidence that training‐induced reductions in SPA may be exacerbated under hypoxic living conditions (Orsi et al., 2025). Finally, as a third block of analysis, we examined whether the LHTL model could alter the concentrations of specific white blood cell subpopulations, given that redox imbalances may also act as signals for triggering inflammatory responses (Bakonyi & Radak, 2004; Dosek et al., 2007; Eltzschig & Carmeliet, 2011; Niess et al., 1999; Pham et al., 2021; Ranneh et al., 2017).

## 2 MATERIALS AND METHODS

### 2.1 Animal care

Forty adult male mice (C57BL/6J) were utilized. During the experimental period, the mice were kept in a room with a controlled environment, including temperature (23 ± 1°C), relative humidity (45%–55%), noise (< 80 decibels) and a 12‐h light/dark cycle (illumination from 6:00 h to 17:59 h). Mice were kept in commercial polyethylene cages (length: 40 cm, width: 33 cm and height: 16 cm; floor area of 1320 cm^2^) and received ad libitum access to commercial standard chow (Nuvilab® CR‐1; Nuvital, BR). Weaned mice were obtained from the animal facility of the University of Campinas, located in the Multidisciplinary Center for Biological Research involving Laboratory Animals (CEMIB). The animals were maintained in a local animal care facility under standard conditions until they reached adulthood. Taking into consideration that chronic social isolation alters metabolic and behavioral parameters of rodents (Benfato et al., 2022; Goldsmith et al., 1978; Moore, 1968; Parker & Morinan, 1986; Schipper et al., 2020; Sharp et al., 2002; Sun et al., 2014; Weiss et al., 2004), the mice were housed collectively (10 mice *per* cage). Procedures were approved by an ethical review committee (Comissão de Ética no Uso de Animais‐ CEUA‐UNICAMP, protocol number 5509–1/2020). All experiments were conducted in accordance with relevant guidelines and regulations such as the European Convention for the Protection of Vertebrate Animals used for Experimental and Other Scientific Purposes (Guideline, 2006) and the National Research Council Guide for the Care and Use of Laboratory Animals (Guideline, 2011).

### 2.2 Study design and hypoxia conditions

**FIGURE 1:**
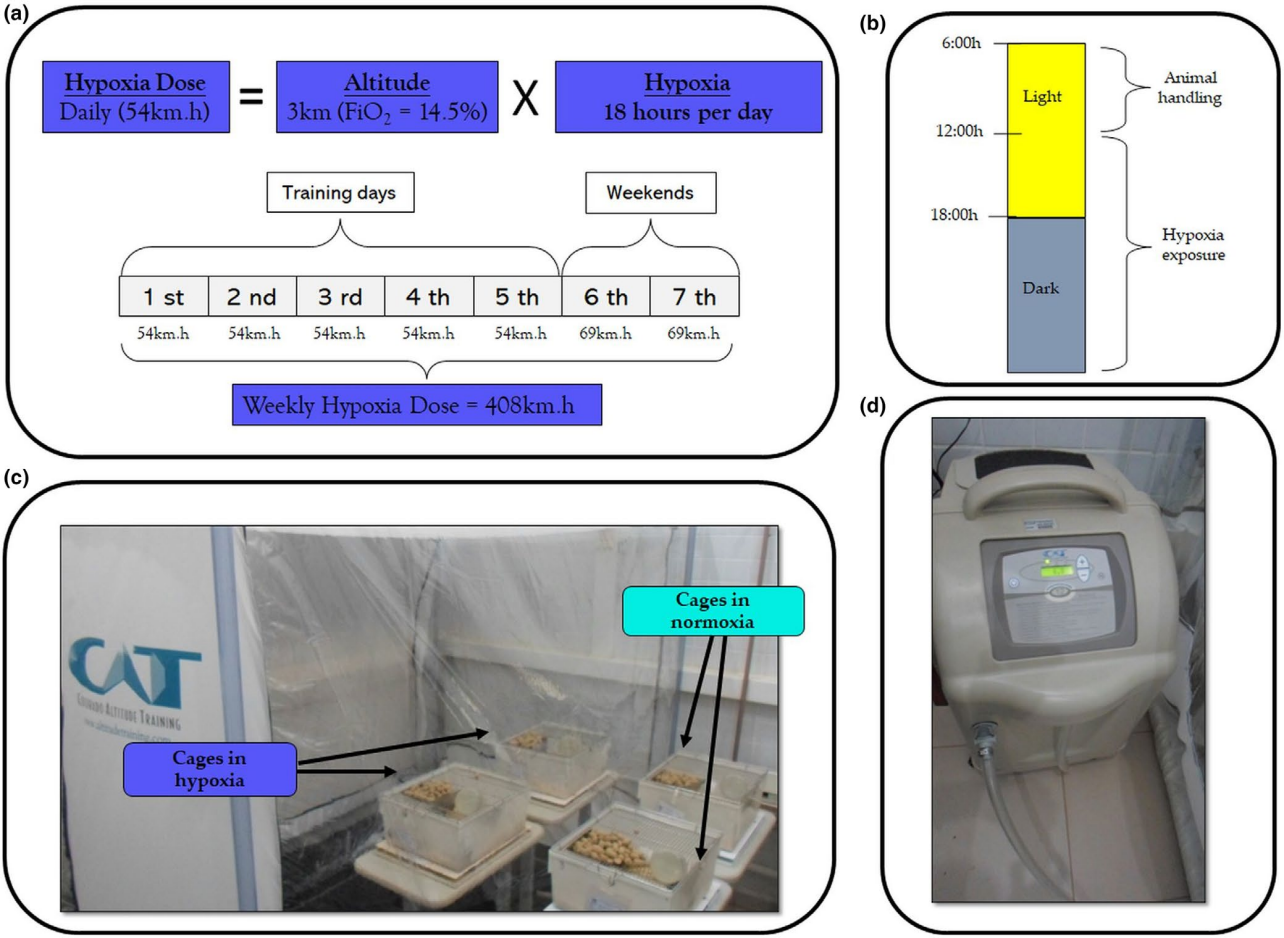
Panel (a) describes a way to measure the hypoxia dose taking into consideration the exposure time (hours) and altitude (km) (Garvican‐Lewis et al., 2016). Mice were exposed to an FiO_2_ = 14.5% to simulate an altitude of approximately 3000 m with exposure lasting 18 h·day^−1^, the daily hypoxia dose corresponded to 54 km·h, as shown in panel (a). On the 2 days per week when no training occurred, the mice were exposed to hypoxia for 23 h per day. During these nontraining days (weekends), animals were in normoxic conditions (for 1 h) only when handled for cage cleaning or weighing. The weekly hypoxia dose reached 408 km·h, and over the 8‐week experiment, the accumulated hypoxia dose totaled 3264 km·h. Panel (b) shows that the hypoxia‐exposed groups were kept in a normobaric tent from 12 pm to 6 am. The panel (c) depicts how the normoxic (NOR) and hypoxic (HYP) cages were positioned, with the HYP cages placed inside the tent (Colorado Altitude Training®, Louisville, Colorado, USA) and the NOR cages outside. Finally, Panel d indicates that hypoxia was induced using a hypoxic gas generator (Panel d).

Rodents were randomly assigned to two environmental conditions: normoxia (NOR) and hypoxia (HYP). Each condition was further divided into nontrained (N) and trained (T) groups, resulting in four experimental groups (N‐NOR, T‐NOR, N‐HYP, T‐HYP), each containing ten rodents. Animal handling occurred in normoxia (FiO_2_ = 19.5%, from 6:00 h to 12:00 h), with oxygen levels monitored using a portable analyzer (Senko SP2nd O_2_). HYP groups were kept in a normobaric‐hypoxic tent (Colorado Altitude Training, USA) for 18 h daily over 8 weeks. HYP groups breathed an inspired oxygen fraction [FiO_2_] of 14.5% to simulate an altitude of 3000 m (Parati et al., 2018). After 8 weeks (at the end of the interventions), all mice were euthanized (cervical dislocation) and subsequently had their tissues collected, as described in detail later in the Collection of Biological Material section. The hypoxia dose as calculated as the product of exposure duration (in hours) and altitude (in kilometers) (Garvican‐Lewis et al., 2016). The total hypoxia dose throughout the 8‐week experiment was 3264 km·h, as illustrated in Figure 1.

### 2.3 Critical velocity (CV) determination

Mice underwent testing to determine their individual CV. Before testing, mice underwent a three‐day acclimation period to treadmill exercise, in accordance with previous protocols (Scariot et al., 2021). The animals performed four running tests on different days, each time running at a steady speed until they were exhausted. Exercise intensities were individually selected to ensure that the time to exhaustion was between 1 and 15 min, as previously recommended (Manchado‐Gobatto et al., 2010; Scariot et al., 2019). Time to exhaustion was defined as the point at which mice were no longer able to sustain coordinated running on the treadmill, even after gentle encouragement by the researcher. No electrical stimuli were employed; instead, motivation was provided by soft tapping with a brush. For each of the four constant‐speed trials, the total distance covered and the corresponding time to exhaustion were recorded. These data points were then plotted on a distance‐versus‐time graph, and linear regression analysis was applied. The CV was determined as the slope (i.e., angular coefficient), representing the highest constant velocity the animal could theoretically sustain over time (Gaesser & Poole, 1996; Hill et al., 2011; Jones et al., 2010). It has also been proposed that CV defines the lower limit of the severe intensity exercise domain, positioned slightly above the anaerobic threshold (Gaesser & Poole, 1996). All tests were conducted under normoxic conditions.

### 2.4 Aerobic training

Training sessions lasted 40 min and were scheduled five times a week, as in a previous study (Scariot et al., 2019). Regarding the training intensity, the mice ran at 80% of the CV, which is within the heavy intensity domain (Gaesser & Poole, 1996). Training was carried out in normoxic conditions. During each training session, the mice engaged in both warm‐up and cool‐down activities. Nontrained groups were acclimatized to the running task to minimize the confounding effects of training. Training volume (in seconds) was monitored in each session to evaluate whether the trained animals exhibited any impairment in their ability to complete the planned training protocol. For example, if the daily session lasted 2400 s (i.e., 40 min), each animal was expected to accumulate a weekly volume of 12,000 s over five training sessions. Over the course of 6 weeks (excluding the weeks used for critical velocity assessment), the expected cumulative training volume would be 72,000 s.

### 2.5 Measurement of spontaneous physical activity (SPA)

SPA was measured by the gravimetric principle using load cells as described previously (Beck et al., 2016; Biesiadecki et al., 1999; Scariot et al., 2019, 2021). SPA includes a range of movements that are not part of planned exercise. In the case of laboratory rodents, SPA represents all the behaviors they naturally perform within the cage environment throughout the day and may include ambulation, grooming, fidgeting, rearing, sniffing, digging, nesting, climbing, general exploration, food‐seeking behaviors, and social interactions which in turn are considered low‐intensity activities. We measured SPA on a per‐cage basis without removing animals from their home cage, capturing all major and sensitive movements such as ambulation, grooming, rearing, and fidgeting (Garland Jr. et al., 2011). The SPA was calculated using Matlab® software following the mathematical formulation previously described (Scariot et al., 2019). SPA was recorded throughout the study (over the course of 8 weeks) at an acquisition frequency of 200 Hz for 18 continuous hours per day. Activity levels were reported separately for the periods of light (from 12:00 to 17:59) and darkness (from 18:00 to 5:59).

### 2.6 Collection of biological material

Euthanasia was performed on mice after an overnight fast, 48 h after the last exercise exposure. Cervical dislocation was selected as the primary euthanasia method, followed immediately by decapitation to ensure complete cessation of brain activity. According to current recommendations, cervical dislocation is considered an acceptable physical method for small‐bodied rodent species such as C57BL/6 mice (Guideline, 2011; Shomer et al., 2020). The technique was carried out by an experienced researcher to ensure rapid and accurate execution, thereby preventing incomplete dislocation and minimizing the risk of unnecessary suffering in the rodents. In addition, the use of cervical dislocation eliminates the need for anesthetic agents, which can alter metabolic variables (Chen et al., 2020; Giroux et al., 2016; Overmyer et al., 2015). Consequently, employing a nonpharmacological euthanasia method ensures that the experimental endpoints reflect the animals' true physiological state without introducing confounding interpretations. Tissues (soleus muscle, white gastrocnemius muscle and hypothalamus) were harvested and frozen in liquid nitrogen and stored at −80°C.

### 2.7 Leukogram analyses

Blood was collected after decapitation into heparinized plastic tubes containing EDTA. The blood was gently homogenized. Analyses were carried out by biomedical professionals and samples were processed in a Sysmex XS‐800i hematology analyzer (Sysmex Europe GmbH, Germany). The concentrations of the following white blood cell types were determined: lymphocytes, monocytes, neutrophils, eosinophils and basophils.

### 2.8 Western blot analyses

Samples were homogenized (on ice) using pestles in a buffer composed of Tris HCl, NaCl, EDTA, IGEPAL, Deoxycholate, SDS, of protease inhibitors at a concentration of 1% (Protease Inhibitor Cocktail, cat# P8340, Sigma‐Aldrich®), and phosphatase inhibitors at a concentration of 1% (Phosphatase Inhibitor Cocktail Set II, cat# US1524625‐1SET, Calbiochem®). Samples were also subjected to sonication to ensure cell lysis (Q55 sonicator, Qsonica®, USA). Protein concentration was measured using the Bradford assay, according to the manufacturer's instructions (Bio‐Rad Protein Assay Dye Reagent Concentrate, cat# 5000006, Bio‐Rad Laboratories). Samples were mixed with LDS sample buffer (with 1% mercaptoethanol), heated at 94°C for 10 min, and loaded (40 μg of protein per lane) in a 10% SDS‐PAGE gel (Mini‐PROTEAN® TGX™ Precast Gel, cat#4561036, Bio‐Rad Laboratories). Electrophoresis was performed with Tris‐glycine buffer, with a constant voltage (130 v) for 1 h. After electrophoresis, gels were briefly washed in Towbin buffer for 5 min. Towbin buffer was comprised of 25 mM of Tris, 192 mM of glycine and 20% of methanol. Proteins were transferred to PVDF membranes (Invitrogen™, iBlot™ 2 Transfer Stacks, cat# IB24002) using the iBlot™ 2 Gel Transfer Device (20 V for 7 min, cat#IB21001). This dry transfer system provides rapid and highly efficient protein transfer, contributing to consistent blot resolution and minimizing potential sources of technical variability. Membranes were stained with a total protein stain (REVERT™ Total Protein Stain, cat# 926–11,010, LI‐COR) and scanned using the 700 nm channel of the Odyssey Fc System (LI‐COR Biosciences, Lincoln, NE, USA). After this, membranes were blocked with 1% milk‐PBS (Nonfat dry milk, cat# 9999, Cell Signaling Technology®) for 1 h at room temperature. Regarding primary antibodies, the membranes were incubated with 5% milk in PBS‐Tween for 1 h at room temperature (OXR1, cat# 13514‐1‐AP, Proteintech) or overnight at 4°C (HIF‐1α alpha antibody, cat# NB100‐479, Novus Biologicals and PGC‐1α alpha antibody, cat# NBP1‐04676, Novus Biologicals). Antibody binding was detected using Goat anti‐Rabbit IgG H&L (IRDye 800CW) preadsorbed (cat# ab216773) at a 1:20,000 dilution for 1 h at room temperature. Fluorescence was detected at 800 nm with the Odyssey Fc System (LI‐COR Biosciences, Lincoln, NE, USA). Both target proteins and total protein were analyzed using near‐infrared fluorescence detection with the Odyssey Fc LI‐COR system. In addition to avoiding interference from the visible light spectrum, this system offers a wide linear dynamic range (LDR; spanning over six orders of magnitude), enabling accurate and reliable quantification of both low‐ and high‐abundance proteins. This capability minimizes issues related to faint signals, exposure variability, and signal saturation. All these technical advantages inherent to the instrumentation alone would already be sufficient to provide more accurate signal detection than traditional chemiluminescence‐based methods; however, we went further by normalizing protein expression using a total‐protein stain. The total‐protein stain was not used only for visual assessment of gel loading but was quantitatively incorporated into the analysis to calculate a precise lane‐normalization factor for each sample. This strategy offers substantial advantages over normalization based on endogenous housekeeping proteins (e.g., GAPDH, β‐actin, tubulin) (Bettencourt et al., 2020; Kirshner & Gibbs, 2018; Maloy et al., 2022). Moreover, total‐protein normalization allows integration of data obtained across different membranes, thereby enhancing statistical robustness and ensuring greater scientific rigor.

### 2.9 Statistical analysis

Statistical processing of the data was performed using the STATISTICA software, version 10 (StatSoft, Inc. (Tulsa, USA, version 7 StatSoft, Inc.)) and to generate graphs we used GraphPad Prism software (version 8.2.1). The assumptions of normality and homogeneity were tested by Shapiro–Wilk and Levene, respectively. Biological variables were analyzed using a two‐way ANOVA to compare the effects of training (nontrained vs. trained) and environment (Normoxia vs. Hypoxia). Another two‐way ANOVA was also conducted to verify whether the training groups exhibited changes in the completed training volume over the weeks (1, 2, 3, 5, 6, and 7). The Fisher LSD posthoc test was used regardless of the presence of significant main or interaction effects. In all cases, the significance level was set at *p* < 0.05.

## 3 RESULTS

**FIGURE 2:**
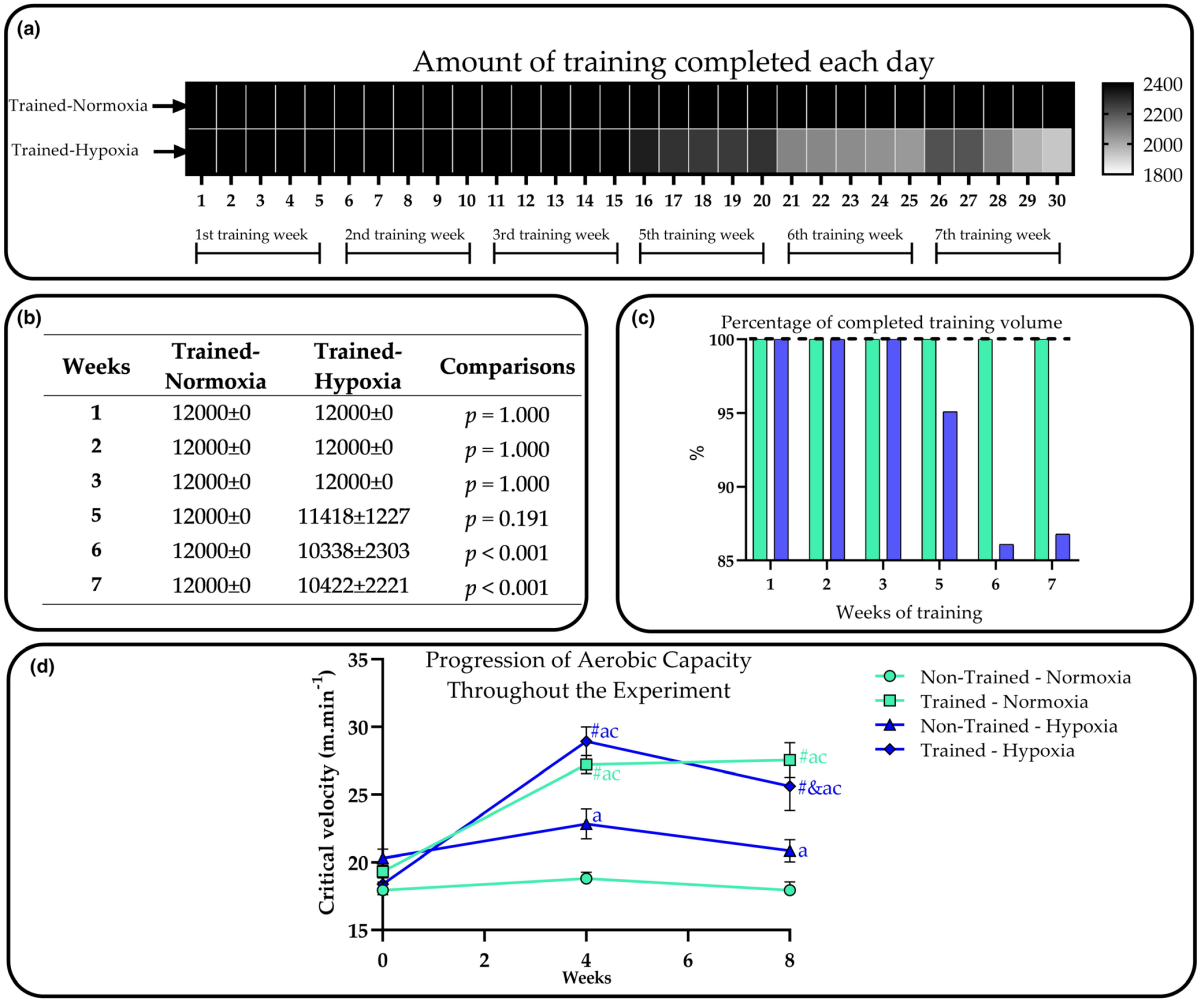
The amount of training completed (in seconds) by the groups maintained under normoxia (NOR) and hypoxia (HYP) conditions is illustrated in the heat map (Panel a), showing daily values. Weekly accumulated training volume (in seconds) is presented in Panel (b). Panel (c) displays the percentage of the planned training volume that was completed, with green representing the NOR group and blue the HYP group. In addition to illustrating the progression of aerobic capacity over the course of the experiment, the critical velocity shown in Panel (d) also provides an indication of training intensity. Considering that the training was set at 80% of the critical velocity, the intensity during the first 3 weeks averaged approximately 14 m·min^−1^ for both trained groups. Following the reassessment conducted in week 4, the training intensity was increased to comply with the overload principle, reaching values close to 22 m·min^−1^ for both groups. Data are exhibited as mean and standard deviation. Statistical analysis: A and c: Significant differences (*p* < 0.05) in relation to N‐NOR, N‐HYP within same week, respectively. # and & significant differences (*p* < 0.05) in relation to baseline and 4 week within same group.

The training volume completed by the groups maintained in normoxia and hypoxia is shown in Figure 2. ANOVA revealed significant effects of the “week” factor (*F* = 3.2, *p* = 0.009), indicating a progressive reduction in training volume during the final weeks of the experiment. This reduction was primarily driven by the animals kept in hypoxia, as evidenced by a significant interaction effect. This finding is consistent with the post hoc comparisons (Figure 2), which showed a significant decrease in training volume during weeks 6 and 7 for the T‐HYP group. Regarding critical velocity, the post hoc comparisons are presented in Panel D of Figure 2. As a main finding, the data suggest that after 3 weeks of training, aerobic capacity increased in mice regardless of the environment in which they lived. However, the group exposed to the “live high–train low” model (T‐HYP group) did not maintain their critical velocity.

**FIGURE 3:**
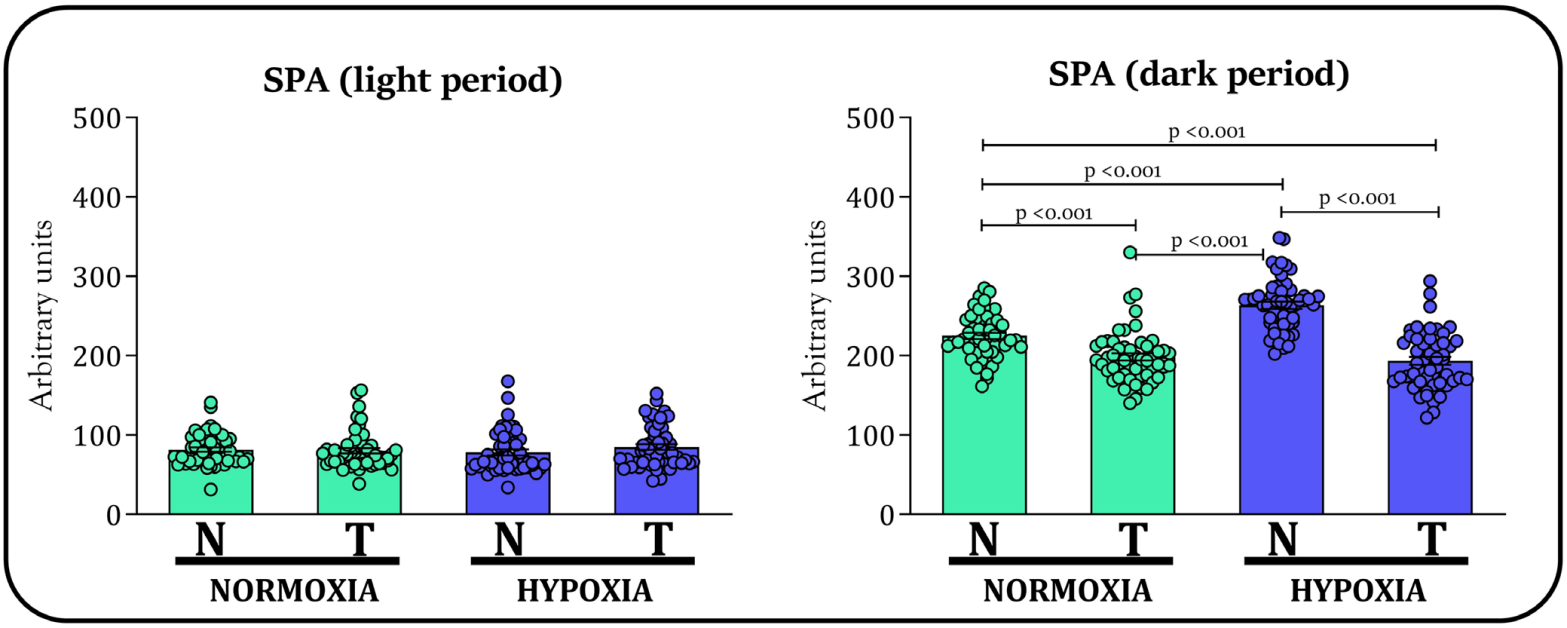
Daily records of SPA for nontrained (N) and trained (T) groups kept in normoxia (NOR) and hypoxia (HYP) over the course of the study. This analysis encompasses all data collected throughout the entire 8‐week period, totaling 52 experimental days. Bars are exhibited as M and SEM.

As detailed in Figure 3, the SPA in the dark period over the entire 8‐week period was higher for the HYP‐groups than NOR‐groups (*F* = 12.9, *p* < 0.001). According to ANOVA, SPA in the dark period was lower in trained groups when compared to nontrained groups, considering the entire 8‐week period (*F* = 110.9, *p* < 0.001). The effect of training in reducing SPA in dark period was more pronounced in mice kept in hypoxia, as shown by a significant interaction between training and environment (*F* = 22.62, *p* < 0.001). Regarding post hoc comparisons, the N‐NOR group exhibited significantly higher SPA in dark period compared to both the T‐NOR (*p* < 0.001), and T‐HYP (*p* < 0.001) groups. SPA in dark period was significantly higher in the N‐HYP group compared to the N‐NOR (*p* < 0.001), T‐NOR (*p* < 0.001), and T‐HYP (*p* < 0.001) groups. Regarding SPA during the light period, no significant effects were found for training (*F* = 0.6, *p* = 0.418), environment (*F* = 0.0, *p* = 0.878), or their interaction (*F* = 1.5, *p* = 0.220). No differences were detected by the post hoc analysis for SPA in the light period.

**FIGURE 4:**
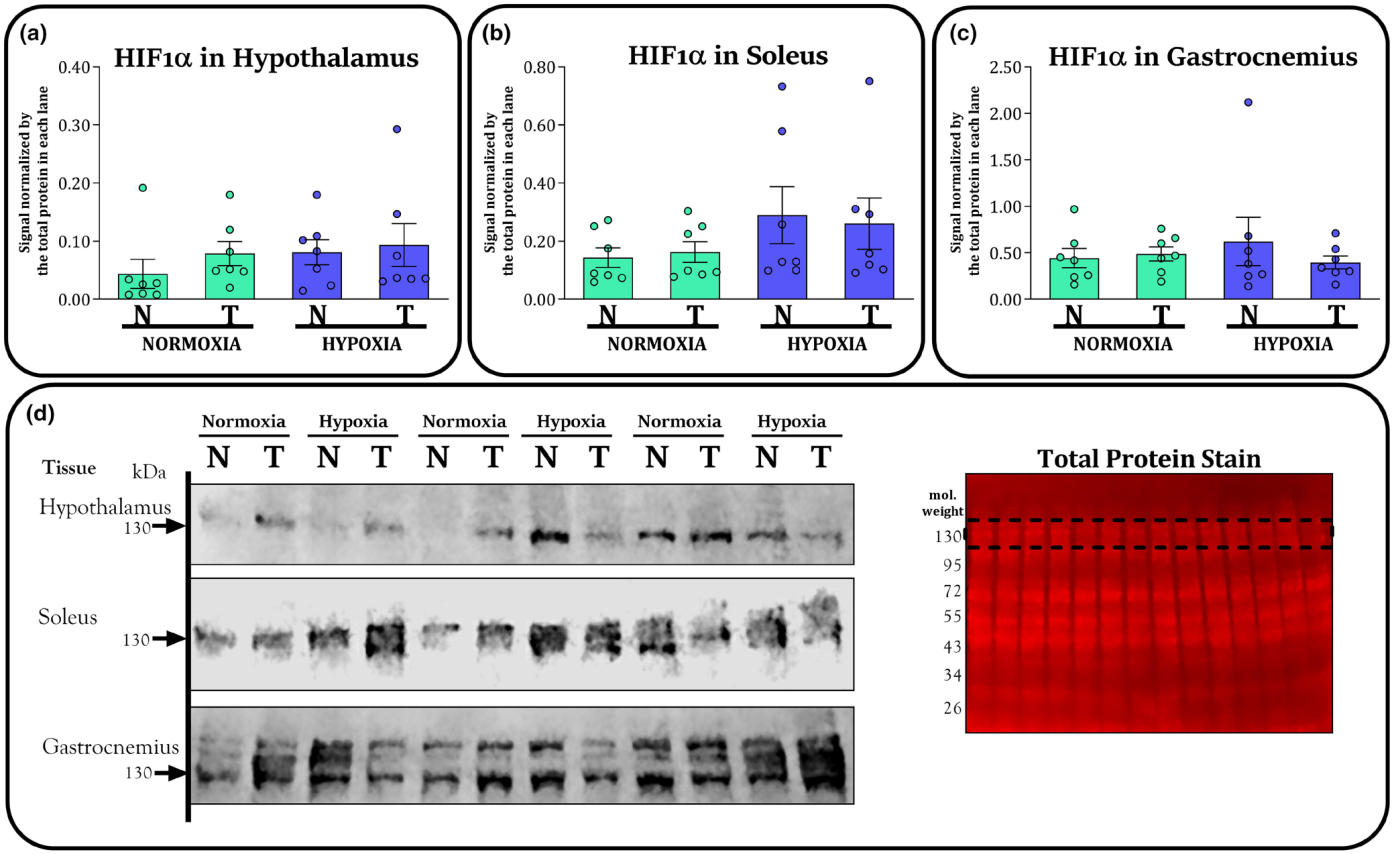
Protein content of HIF‐1α in the hypothalamus (panel a), soleus muscle (panel b), white gastrocnemius muscle (panel c) and for nontrained (N) and trained (T) groups kept in normoxia (NOR) and hypoxia (HYP) at the end of study. Panel d shows a representative image confirming the detection of the target protein bands scanned at 800 nm using near‐infrared fluorescence detection. The same membranes were stained for total protein and scanned at 700 nm to normalize for interlane differences in protein loading. The dashed lines in the total protein stain indicate the molecular weight range corresponding to the region in which the target protein bands were detected. Bars are exhibited as M and SEM (*n* = 7 per group).

Figure 4 exhibits the western blot results of HIF‐1α in hypothalamus (panel A), soleus muscle (panel B), and white gastrocnemius muscle (panel C). There was no significant effect of training (*F* = 0.7; *p* = 0.384) or environment (*F* = 0.93; *p* = 0.343) on HIF‐1α in the hypothalamus. Regarding the soleus, HYP‐groups tended to have higher HIF‐1α than NOR‐groups, according to the ANOVA (*F* = 3.0; *p* = 0.095). There was no significant effect of training on HIF‐1α in the soleus (*F* = 0.0; *p* = 0.943). There was no significant effect of training (*F* = 0.3; *p* = 0.551) or environment (*F* = 0.0; *p* = 0.773) on HIF‐1α in the white gastrocnemius muscle. We found no significant interactions on HIF‐1α in the hypothalamus (*F* = 0.1; *p* = 0.671), soleus (*F* = 0.1; *p* = 0.73), or white gastrocnemius muscle (*F* = 0.8; *p* = 0.372).

**FIGURE 5:**
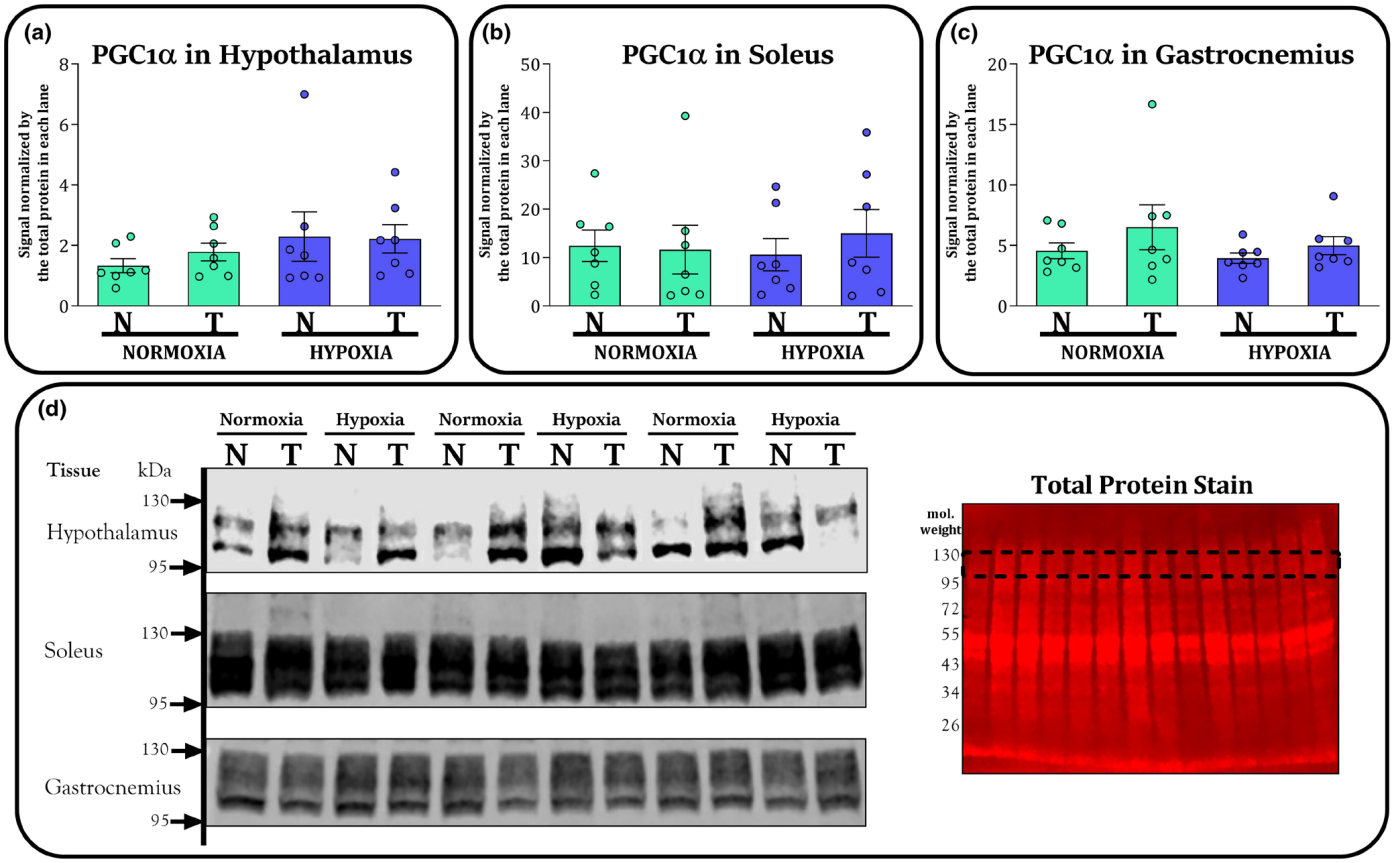
Protein content of PGC‐1α in the hypothalamus (panel a), soleus muscle (panel b), white gastrocnemius muscle (panel c) for nontrained (N) and trained (T) groups kept in normoxia (NOR) and hypoxia (HYP) at the end of study. Panel d shows a representative image confirming the detection of the target protein bands scanned at 800 nm using near‐infrared fluorescence detection. The same membranes were stained for total protein and scanned at 700 nm to normalize for interlane differences in protein loading. The dashed lines in the total protein stain indicate the molecular weight range corresponding to the region in which the target protein bands were detected. Bars are exhibited as M and SEM (*n* = 7 per group).

PGC‐1α western blot results in hypothalamus (panel A), soleus muscle (panel B), and white gastrocnemius muscle (panel C) are exhibited in Figure 5. There was no significant effect of training (*F* = 0.1; *p* = 0.714) or environment (*F* = 1.8; *p* = 0.185) on PGC‐1α in the hypothalamus. Additionally, there was no significant effect of training (*F* = 0.1; *p* = 0.674) or environment (*F* = 0.0; *p* = 0.861) on PGC‐1α in the soleus. There was no significant effect of training (*F* = 1.9; *p* = 0.178) or environment (*F* = 1.0; *p* = 0.324) on PGC‐1α in the white gastrocnemius muscle. We found no significant interactions on PGC‐1α in the hypothalamus (*F* = 0.2; *p* = 0.607), soleus (*F* = 0.3; *p* = 0.543), or white gastrocnemius muscle (*F* = 0.1; *p* = 0.677).

**FIGURE 6:**
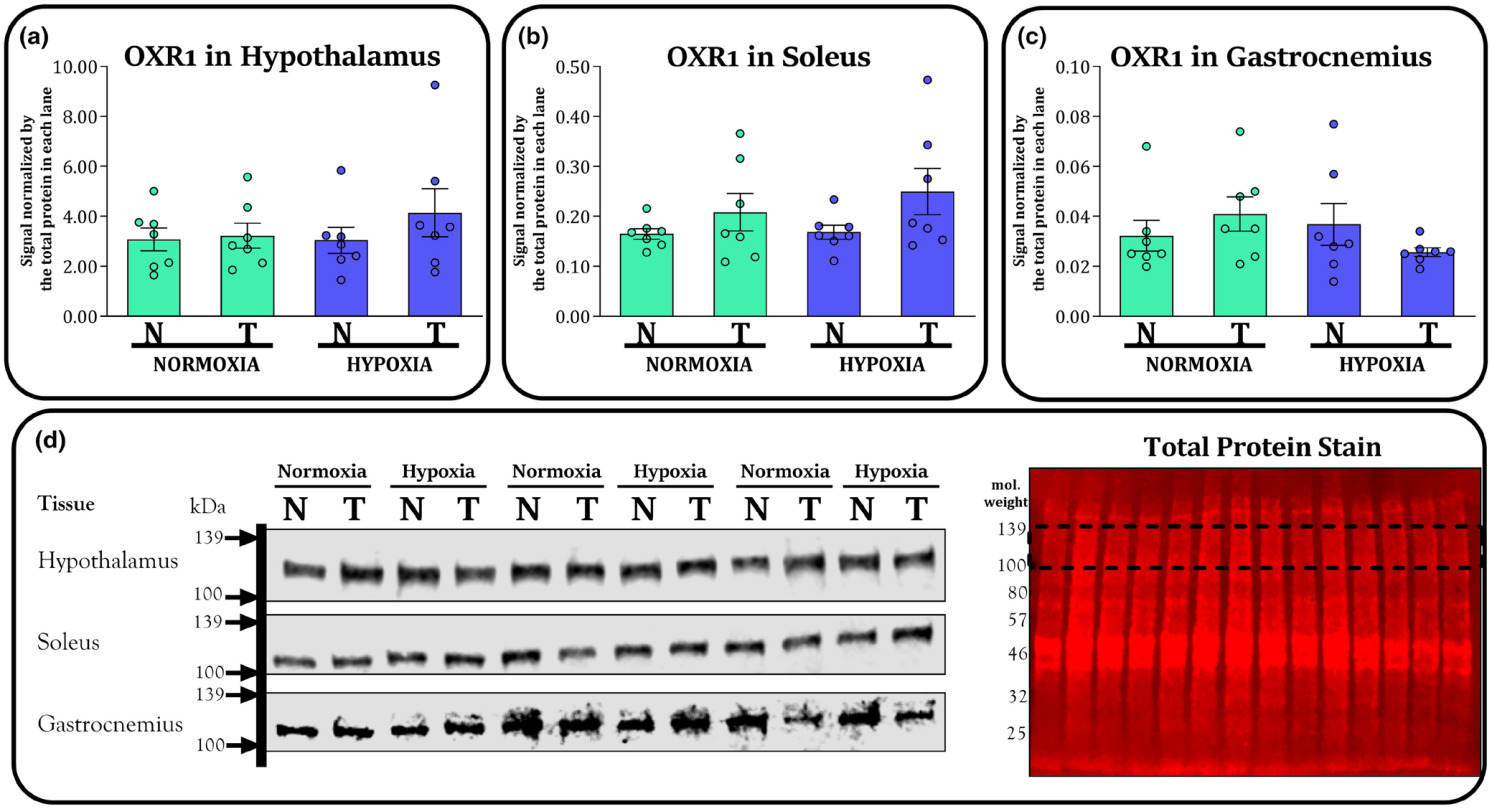
Protein content of OXR1 in the hypothalamus (panel a), soleus muscle (panel b), white gastrocnemius muscle (panel c) for nontrained (N) and trained (T) groups kept in normoxia (NOR) and hypoxia (HYP) at the end of study. Panel d shows a representative image confirming the detection of the target protein bands scanned at 800 nm using near‐infrared fluorescence detection. The same membranes were stained for total protein and scanned at 700 nm to normalize for interlane differences in protein loading. The dashed lines in the total protein stain indicate the molecular weight range corresponding to the region in which the target protein bands were detected. Bars are exhibited as M and SEM (*n* = 7 per group).

Figure 6 exhibits the western blot results of OXR1 in the hypothalamus (panel A), soleus muscle (panel B), and white gastrocnemius muscle (panel C). Post hoc analyses did not detect significant differences between groups; however, trained groups tended to have higher OXR1 in soleus than nontrained groups, according to the ANOVA results (*F* = 4.0; *p* = 0.055). There was no significant effect of training on OXR1 in the white gastrocnemius muscle (*F* = 0.0; *p* = 0.825) and hypothalamus (*F* = 0.9; *p* = 0.337). We found no significant effect of the environment on OXR1 in the soleus (*F* = 0.5; *p* = 0.476), white gastrocnemius muscle (*F* = 0.7; *p* = 0.399), or hypothalamus (*F* = 0.4; *p* = 0.498). We found no significant interactions on OXR1 in soleus (*F* = 0.3; *p* = 0.544), white gastrocnemius muscle (*F* = 2.4; *p* = 0.129), and hypothalamus (*F* = 0.5; *p* = 0.463).

Results of the white blood cells profile are presented in Table 1. ANOVA indicated that nontrained groups had significantly lower counts of leukocytes, lymphocytes, eosinophils, and basophils (all expressed in mm^3^) compared to trained groups. Interestingly, a significant interaction revealed that the training‐induced reduction in leukocyte and lymphocyte counts occurred only in mice housed under normoxic conditions, but not in those housed in hypoxia. Conversely, the significant interaction detected by ANOVA showed that the training‐induced reduction in eosinophil counts occurred exclusively in mice housed under hypoxic conditions, with no significant effect observed in normoxia. Post hoc analysis revealed that the N‐HYP group exhibited the highest eosinophil counts among all groups. As detected by the effect of the environment, hypoxia exposure led to a higher percentage of lymphocytes and lower monocyte counts compared to normoxia groups. Detailed results of the two‐way ANOVA are provided in Table 1.

**TABLE 1:** Leukocytes, lymphocytes, monocytes, neutrophils, eosinophils, and basophils for nontrained (N) and trained (T) groups kept in normoxia (NOR) and hypoxia (HYP) at the end of study.

	Normoxia		Hypoxia		ANOVA		
	N	T	N	T	Training (N vs. T)	Environment (NOR vs. HYP)	Interaction
Leukocytes (mm^3^)	8999.0 ± 2326.0	3990.0 ± 1310.5a	5522.0 ± 2228.3a	5101.0 ± 1814.8a	*F* = 18.3 *p* < 0.001*	*F* = 3.4 *p* = 0.070	*F* = 13.1 *p* < 0.001*
Lymphocytes (mm^3^)	7533.5 ± 1768.6	3225.9 ± 1052.3a	4935.1 ± 2098.9ab	4618.2 ± 1815.2a	*F* = 17.1 *p* < 0.001*	*F* = 1.1 *p* = 0.287	*F* = 12.7 *p* = 0.001*
Lymphocytes (%)	84.8 ± 8.1	81.5 ± 10.5	89.0 ± 6.2	89.6 ± 8.4	*F* = 0.2 *p* = 0.616	*F* = 5.2 *p* = 0.027*	*F* = 0.5 *p* = 0.478
Monocytes (mm^3^)	902.4 ± 868.4	523.1 ± 519.1	235.0 ± 253.6a	164.6 ± 269.2a	*F* = 1.6 *p* = 0.201	*F* = 8.8 *p* = 0.005*	*F* = 0.7 *p* = 0.377
Monocytes (%)	9.1 ± 8.2	12.6 ± 10.9	4.7 ± 5.3b	4.2 ± 8.2b	*F* = 0.3 *p* = 0.575	*F* = 5.7 *p* = 0.021	*F* = 0.5 *p* = 0.465
Neutrophils (mm^3^)	147.9 ± 124.6	93.7 ± 143.8	93.7 ± 78.0	99.8 ± 67.2	*F* = 0.4 *p* = 0.487	*F* = 0.4 *p* = 0.487	*F* = 0.7 *p* = 0.385
Neutrophils (%)	1.8 ± 1.4	3.2 ± 3.8	1.8 ± 1.4	1.9 ± 1.2	*F* = 1.0 *p* = 0.304	*F* = 0.8 *p* = 0.373	*F* = 0.9 *p* = 0.344
Eosinophils (mm^3^)	4.5 ± 7.8	3.3 ± 6.9	18.8 ± 18.6acd	2.0 ± 4.2	*F* = 6.5 *p* = 0.014*	*F* = 3.4 *p* = 0.073	*F* = 4.9 *p* = 0.032*
Eosinophils (%)	0.04 ± 0.1	0.13 ± 0.3	0.34 ± 0.3abd	0.04 ± 0.1	*F* = 2.4 p = 0.129	*F* = 2.4 *p* = 0.129	*F* = 8.7 *p* = 0.005*
Basophils (mm^3^)	407.4 ± 247.6	196.8 ± 96.3a	235.5 ± 149.0a	216.1 ± 130.4a	*F* = 4.6 *p* = 0.038*	*F* = 2.0 *p* = 0.163	*F* = 3.1 *p* = 0.083
Basophils (%)	4.3 ± 1.8	4.8 ± 1.4	4.1 ± 1.8	4.3 ± 2.0	*F* = 0.4 *p* = 0.512	*F* = 0.4 *p* = 0.512	*F* = 0.0 *p* = 0.758

*Note*: Values are expressed relative to leukocytes (%). Data are in mean and standard deviation (*n* = 10 per group). Statistical analysis: a, b, c and d: significant differences (*p* < 0.05) in relation to N‐NOR, T‐NOR, N‐HYP and T‐HYP, respectively. The symbol “asterisk” indicates a significant effect or interaction.

## 4 DISCUSSION

**FIGURE 7:**
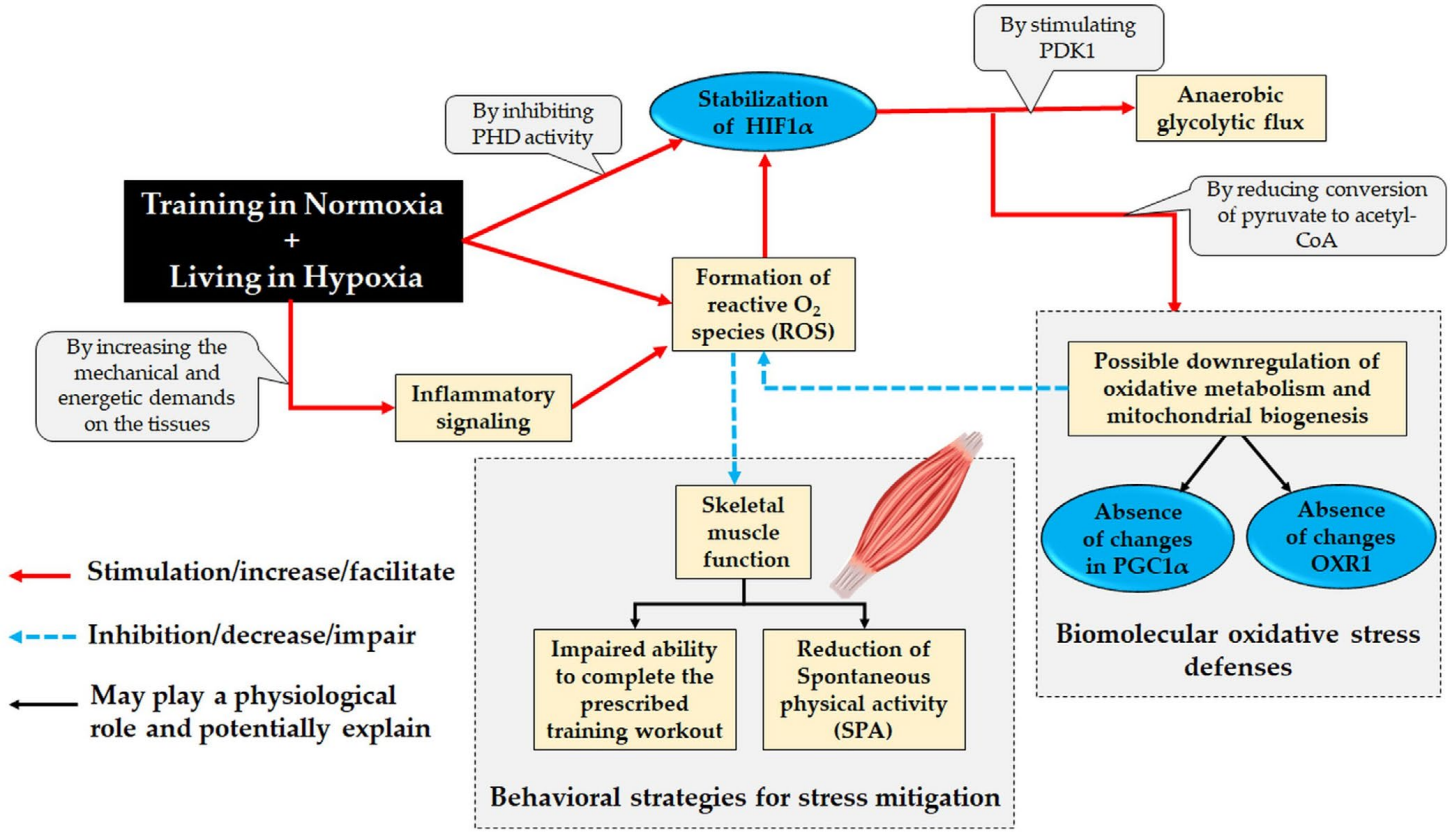
Schematic representation of potential physiological interactions between aerobic training and hypoxia discussed in the present study. Although not observed at the time of analysis, it is likely that stabilization of HIF‐1α occurred during the period of hypoxic exposure. Inhibition of prolyl hydroxylase (PHD) activity can enhance anaerobic glycolytic flux and downregulate oxidative metabolism. This metabolic shift may serve as a strategy to reduce reactive oxygen species generation. The LHTL model may have subjected skeletal muscle to heightened stress, as mice exposed to this condition—unlike those trained while living in normoxia—exhibited a pronounced reduction in spontaneous physical activity (SPA) and a diminished ability to complete the prescribed training sessions. While this may appear causal from a statistical viewpoint, it is important not to disregard the possibility of a regulatory central mechanism whereby a signal is sent for the muscle to reduce its activity in order to prevent damage, even in the absence of observable changes in molecular markers typically associated with oxidative stress mitigation, such as PGC‐1α and OXR1.

Our study contributes to the understanding of how *living high–training low* affects key elements of metabolism such as HIF‐1α and PGC‐1α. The pronounced reductions in SPA and training volume observed in mice exposed to LHTL suggest they experienced greater physiological challenges compared to trained mice kept entirely in normoxia. These induced behavioral changes may have functioned as an adaptive response, helping to alleviate physiological stress and support muscle recovery before the activation of antioxidant pathways such as OXR1. We developed a summary (Figure 7) that highlights the connections among the main variables examined in this study, specifically in relation to hypoxia and aerobic training.

It is appropriate at this point in the discussion to make considerations about HIF‐1α, not only because of its undeniable importance in hypoxia, but also because certain misconceptions have, unfortunately, solidified into a collective perception. A common example is the assumption that every study involving hypoxia should always find an increase in HIF‐1α protein. However, this view is potentially misleading, as the detection and magnitude of HIF‐1α activation depend on multiple factors that extend well beyond mere exposure to hypoxia, as will be discussed below. Therefore, generalizing HIF‐1α findings obtained under specific experimental conditions as universally applicable may lead to conceptually flawed and oversimplified interpretations.

The duration of hypoxic exposure is a critical factor modulating HIF‐1α dynamics, and caution is therefore required when considering this temporal dimension. It is frequently assumed that the HIF‐1α response observed during acute hypoxia will persist during prolonged exposure. However, such extrapolations should not be made, as they are not supported by experimental evidence, which indicates that the acute increase in HIF‐1α protein is inherently transient and not sustained over time. For instance, Le Moine et al. (2011) showed that HIF‐1α and PDK1 protein expression in mice exposed to hypobaric hypoxia for 1 week was markedly lower than that observed after only 24 h of exposure. This reduction was so pronounced that, after 1 week, HIF‐1α and PDK1 levels were virtually indistinguishable from those of the normoxic group, despite the ongoing hypoxic exposure. Chávez et al. (2000) described a similar temporal profile in rats, showing that HIF‐1α rose sharply during the initial phase of hypoxia, as expected, but subsequently decreased by day 21 despite persistently low arterial oxygen tension. Consistent with these in vivo observations, studies in cultured cells demonstrate that HIF‐1α protein levels decline within 1 week of sustained hypoxic incubation (Bartoszewska et al., 2015; Ginouvès et al., 2008; Ravenna et al., 2014; Stiehl et al., 2006). Thus, the absence of an increase in HIF‐1α in our study should not be interpreted as evidence that hypoxic signaling was absent throughout the experimental period. Because mechanisms that attenuate HIF‐1α stabilization are activated already within hours and certainly within days of hypoxic exposure, our findings are biologically coherent, given that tissue sampling occurred only after 8 weeks, well beyond the transient window of HIF‐1α upregulation. Obtaining tissue samples solely to capture a transient increase in HIF‐1α expression during the initial days of hypoxia would be logistically unfeasible; however, future studies should consider the assessment of noninvasive blood markers of erythropoietic activation, such as erythropoietin, as these may provide a more complete understanding of hypoxic adaptations that could not be achieved with the current experimental design.

The decline of HIF‐1α despite continued hypoxic exposure is commonly attributed to HIF‐1α desensitization, an adaptive response considered essential for cell survival. Consistent with this view, Ginouvès et al. (2008) demonstrated that sustained forced activation of HIF‐1α under chronic hypoxia can induce cell death. Although the mechanisms underlying this downregulation are not fully resolved, accumulating evidence implicates prolyl hydroxylase domain (PHD) enzymes as central regulators of this process (Ginouvès et al., 2008; Marxsen et al., 2004; Stiehl et al., 2006). While PHD inhibition is required to ensure HIF‐1α stabilization during acute hypoxia, chronic hypoxic exposure is associated with increased expression of PHDs—particularly the PHD2 and PHD3 isoforms. This adaptive feedback mechanism expands the cellular pool of PHD molecules, enabling rapid HIF‐1α degradation in response to even brief episodes of reoxygenation. When placed in the context of our experimental model, this mechanism provides an additional explanation for the coherence of our HIF‐1α findings, given that episodes of reexposure to normoxic conditions occurred regularly during daily training sessions and, most notably, during tissue harvesting on the day of euthanasia.

Beyond exposure duration, hypoxic intensity is another key determinant of HIF‐1α stabilization. However, the relationship between oxygen levels and HIF‐1α responses depends strongly on the experimental model, and comparisons across in vitro and in vivo studies must be made cautiously. A substantial portion of the classical evidence supporting robust Western blot signals for HIF‐1α originates from in vitro experiments in which cell cultures are exposed to intensely hypoxic conditions, with oxygen levels reduced to ~1% O₂ or lower (Abreu‐Rodríguez et al., 2011; Arsham et al., 2003; Bracken et al., 2006; Ginouvès et al., 2008; Jewell et al., 2001; Marxsen et al., 2004; Namas et al., 2011; Papandreou et al., 2006; Wang et al., 2013). While cell cultures have been helpful in elucidating the molecular machinery of HIF‐1α regulation, they operate under isolated and highly simplified conditions that differ markedly from the integrated and heterogeneous physiological environment of intact organisms. Furthermore, the degree of oxygen deprivation imposed in vitro models (~1% O₂) is rarely encountered by most tissues in vivo and is typically restricted to exceptional contexts, such as severe pathological states (e.g., solid tumors) or exposure to severe environmental hypoxia (Brizel et al., 1995; Koong et al., 2000; McKeown, 2014; Vaupel et al., 2002). This distinction helps explain why in vivo studies reporting marked HIF‐1α stabilization generally rely on more severe hypoxic exposures, often involving inspired oxygen fractions near or below 10% (Belaiba et al., 2007; Chaudhary et al., 2012; De Palma et al., 2007; Le Moan et al., 2015; Stroka et al., 2001; Van Thienen et al., 2016; Xu et al., 2021). While HIF‐1α stabilization is more pronounced under severe environmental hypoxia, this does not mean that HIF‐1α signaling is absent under moderate hypoxic conditions. We speculate that the absence of increased HIF‐1α protein levels in our hypoxic groups is unlikely to be attributable to insufficient hypoxic intensity. Supporting this interpretation, previous studies have reported increases in HIF‐1α in specific tissues under hypoxic conditions (~15% O₂) comparable to those employed in the present study (Jochmans‐Lemoine et al., 2016; Stroka et al., 2001). Moreover, even more severe hypoxia would not necessarily result in sustained HIF‐1α accumulation because of desensitization mechanisms discussed above, and would be poorly tolerated in an 8‐week training protocol. Therefore, the use of a moderate hypoxic stimulus (FiO₂ = 14.5%) was both physiologically appropriate and consistent with previous chronic rodent studies demonstrating adaptive responses (Bor‐Kucukatay et al., 2014; Çolak et al., 2021; Shi et al., 2013). Human studies further support the notion that altitudes between 2000 and 3000 m are optimal (Chapman et al., 2014; Wilber et al., 2007), which is consistent with the FiO₂ of 14.5% used in the present study to approximate an altitude of ~3000 m (Parati et al., 2018). Our choice of this simulated altitude therefore strikes a balance, avoiding extremes that could be either ineffective or detrimental. Specifically, lower altitudes (<1500 m) may provide an insufficient hypoxic stimulus, whereas prolonged exposure to very high altitudes (>4000 m) is associated with increased physiological dysfunction. As a final consideration, it is important to emphasize that the absence of detectable HIF‐1α stabilization in an in vivo hypoxia study should not invalidate the investigation or undermine its credibility; on the contrary, it highlights the intrinsic complexity of hypoxic HIF‐1α biology.

Regarding the lack of significant differences in PGC‐1α, this outcome is not surprising. Martinez‐Bello et al. (2011) have similarly failed to demonstrate an additive effect of hypoxia combined with training on PGC‐1α protein levels in muscle tissue of rodents under LHTL model. This might possibly be a result of downregulation of PGC‐1α as a hypoxia‐induced adaptation, which would any improvements elicited by aerobic training. The potential for an interaction between the effects of hypoxia and aerobic training, coupled with the absence of studies evaluating a possible interaction between these two effects, highlights the urgent need for further exploration using the LHTL model. Indeed, even when examining studies that investigate hypoxia in isolation, there is considerable heterogeneity in the evidence regarding PGC‐1α–related responses. While some studies have failed to demonstrate reductions in PGC‐1α in response to hypoxia (Chaillou et al., 2013; Ferri et al., 2018), other investigations have found a downregulation of PGC‐1α as a hypoxia‐induced adaptation (Gamboa & Andrade, 2010; Levett et al., 2012). Even though PGC‐1α is well known for its biological role in regulating aerobic metabolism, mitochondrial biogenesis and muscle atrophy (Knutti & Kralli, 2001; Lin et al., 2002; Popov, 2020; Sandri et al., 2006; Wang et al., 2017; Wu et al., 1999), considerable gaps remain in our understanding of how this pathway responds to specific interventions.

Investigating the modulation of protective proteins (such as OXR1) that safeguard cells and their components, such as DNA, from oxidative damage becomes essential (Zhang et al., 2018). Given this role, it was hypothesized that mice exposed to the LHTL model (i.e., the T‐HYP group) would exhibit increased OXR1 expression compared with the other experimental groups. Contrary to our hypothesis, however, LHTL exposure did not have a significant impact on OXR1 levels. This led us to speculate some possibilities. One possible explanation is that the combination of training in normoxia and living in hypoxia, as applied in the present study, may not have constituted a sufficiently intense physiological stressor to elicit an upregulation of OXR1, whose activation is likely to be more prominent under severe oxidative stress conditions, such as disease, overtraining, or heightened energy demands. Another possibility is that the reduction in SPA and training volume functioned as a compensatory mechanism through which the musculature was able to recover and prevent damage, thereby eliminating the need for changes in OXR1 expression. Furthermore, a possible downregulation of aerobic metabolism while mice are living in hypoxia could impair muscle repair processes, given that recovery is highly dependent on this pathway. This raises an important consideration: is it truly effective to expose animals to hypoxia every day during a critical recovery window that is essential for tissue regeneration? Although a key feature of the LHTL model is the restriction of hypoxia exposure to nontraining periods, it may be necessary to explore adaptations in which hypoxia is avoided on days when recovery is particularly critical. For instance, this could be guided by indicators such as elevated creatine kinase levels or behavioral signs of muscle soreness. However, one point that should be emphasized is that these apparent impairments in completing the training sessions only emerged from the fifth training week onward—after the training intensity was increased. During the first 3 weeks, the execution of the workouts was consistent and complete. This raises another question: why did the hypoxia‐exposed trained mice fail to complete the sessions after the intensity adjustment, even though the new workload (set at 80% of critical velocity) was proportionally the same for all groups? Although we do not yet have a definitive answer, it seems clear that there must be an optimal combination (Which we do not yet know) of training variables for the success of the LHTL model. In terms of physical fitness, it is not possible to conclude that the LHTL model is more advantageous, as the improvements in aerobic capacity did not surpass those observed in mice that both trained and lived in normoxia.

It is suggested in the literature that excessive formation of reactive oxygen species (ROS) can impair muscle function (McGinnis et al., 2014; Reid et al., 1993), however, these effects do not appear to be generalized and may vary depending on muscle type. In this context, the soleus muscle appears to have greater vulnerability to ROS‐mediated oxidative damage compared to the gastrocnemius (Yanar et al., 2019). Based on this, we initially hypothesized that OXR1 expression would be higher in the soleus muscle (but not in the gastrocnemius) of mice exposed to LHTL compared to their respective control counterparts. Although post hoc analysis revealed no statistically significant differences to support this hypothesis, it is noteworthy that the comparison between the T‐HYP and N‐NOR groups yielded a *p*‐value of 0.452 for the gastrocnemius, whereas for the soleus, the *p*‐value was 0.064. This is clearly just a trend, and given the absence of statistically significant differences, any suggestion of greater susceptibility of the soleus to LHTL‐induced stress remains purely speculative and warrants further investigation.

The strong connection between hypoxia, exercise, and immune systems justifies our analysis of leukocyte concentrations in our study. Although there is a relatively large body of evidence from human and animal model studies on leukocyte changes following altitude exposure (Klokker et al., 1993; Thake et al., 2004; Beidleman et al., 2006; Mariggiò et al., 2010; Al‐Sweedan & Alhaj, 2012), many uncertainties remain regarding whether aerobic training‐induced changes in leukocyte concentrations would be potentiated or suppressed by chronic hypoxia exposure. In the present study, total leukocytes (lymphocytes and monocyte) decreased in animals kept in hypoxia (compared to the N‐NOR group). A decline in these cells could indicate that they may have migrated or trafficked from the blood to the tissues (Dhabhar et al., 2012; Rahat et al., 2006; Strehl et al., 2014), but in our context, this interpretation must remain at a speculative level. It is important to remember that monocytes differentiate into macrophages and are recruited to injured sites to aid in host defense. While this process is necessary for healing, an exacerbated inflammatory response may limit the supply of oxygen and nutrients to the tissue. Further, inflammatory cells accumulate in these hypoxic areas, playing a critical role in the release of chemotactic factors (Bosco et al., 2008; Knighton et al., 1983; Lokmic et al., 2012). Given that the accumulation of immune cells in tissues could potentially lead to oxidative stress (Dietrich‐Muszalska et al., 2012; Rodríguez‐Iturbe et al., 2004), creating a cellular environment unfavorable for muscle contractility, it is reasonable to speculate that this mechanism may have contributed to the reduction in training volume observed specifically in the T‐HYP group. However, this interpretation should be approached with caution, since our conclusions were based on systemic (blood) measurements rather than direct assessments of immune cell infiltration or oxidative stress within muscle tissue.

A key finding in our study was the increase in eosinophil levels in the N‐HYP group (nontrained mice exposed to hypoxia). This aligns with existing research showing that low oxygen levels can enhance eosinophil survival (Porter et al., 2017). The hypoxia‐induced increase in eosinophils may represent a beneficial adaptive response aimed at improving oxygen delivery, thereby enhancing performance through the upregulation of pro‐angiogenic factors such as vascular endothelial growth factor (VEGF) (Horiuchi & Weller, 1997; Nissim Ben Efraim et al., 2010). Interestingly, this increase in eosinophils was not seen in the LHTL group (trained mice also exposed to hypoxia). We speculate that aerobic training may counteract the hypoxia‐induced signaling mechanisms that usually upregulate eosinophil survival and recruitment, particularly those involving exotoxins (Horiuchi & Weller, 1997; Nissim Ben Efraim et al., 2010). Based on these results, we believe that immunomodulatory adaptations resulting from the interaction between hypoxia and aerobic training are more complex, and therefore should be more thoroughly investigated, as well as across different sports and exercise modalities, and among a variety of mammalian species.

While carefully conducted, this study has certain limitations. Despite employing a highly sophisticated Western blotting approach, the study remains limited in its ability to provide a deeper characterization of the mechanistic pathways underlying oxidative metabolism and redox regulation. Such pathways could be more comprehensively explored using advanced assessments, including high‐resolution respirometry and detailed mitochondrial assays. In addition, we did not perform in vitro muscle contractility testing, which could have provided complementary information to the in vivo evaluations obtained for exercise‐related parameters and SPA. The absence of these in vitro techniques may have limited our ability to pinpoint the exact origin of the observed impairments, whether they stem from structural alterations of the contractile apparatus or from metabolic changes. Nevertheless, even without fully elucidating the underlying mechanism, our findings clearly demonstrate significant muscle functional impairments in rodents subjected to the LHTL model. This suggests that the challenges posed by the combination of hypoxia (even moderate) and aerobic training under normoxic conditions are substantial and far from trivial.

Another issue that is beyond the scope of this study is that we cannot assert whether the adaptations would have been different if the animals had been subjected to natural high‐altitude environments. From our perspective, our choice to use simulated hypoxia through normobaric tents was appropriate. This method allowed us to create a controlled environment that mimics the conditions of high altitude, enabling us to study the effects of hypoxia without the confounding factors associated with natural high‐altitude settings (such as UV light exposure, temperature variations, and nutritional deficiencies). Since we did not test different doses of hypoxia or intensities and durations of training, our design does not allow us to determine the optimal duration or degree of hypoxia exposure or the optimal training regimen for improving key elements of metabolism. There has already been some discussion among researchers about the individualization of hypoxia dosing (Bertucci et al., 2024; Soo et al., 2020). Certainly, there is discussion among sport physiologists about proper dosing to improve sport performance (Sinex & Chapman, 2015). We predict that, in the near future, individualized hypoxia regimens will be crucial for designing research studies and for implementation of optimized programs to elicit physiological adaptations and improve performance. Training settings are crucial to the efficacy of the LHTL model, and in recognizing this, we individualized exercise intensities according to each mouse's CV. This approach reflects our commitment to recognizing the critical role of training settings in achieving success within the LHTL model. Nonetheless, our research is a preliminary exploration, and many unanswered questions persist.

Indeed, research on the application of the LHTL model in animals is still in its early stages, and to the best of our knowledge, fewer than ten studies have been published to date (Miyazaki & Sakai, 2000; Martinez‐Bello et al., 2011; Li & Wang, 2013; Çolak et al., 2021; Scariot et al., 2023). Key areas for further exploration include comparing the effects of continuous versus interval training and understanding the role of periodization and recovery times in optimizing physiological outcomes. The LHTL model deserves greater scientific attention because it offers a cost‐effective and logistically efficient alternative to traditional high‐altitude training. The simulation of a hypoxic environment (altitude chambers/tents) makes it easier for athletes to use hypoxia models everywhere, avoiding some inconveniences, such as immune suppression and sleep disturbances that may limit athletic performance (Flaherty et al., 2016; Pyne et al., 2000). Although the primary interest in the application of hypoxia continues to reside in the fields of exercise physiology and sport science (Viscor et al., 2018), a deeper understanding of hypoxic responses in aerobically trained organisms is essential, given that these adaptations extend beyond performance and carry important implications across biological and medical disciplines.

## 5 CONCLUSION

Under the conditions implemented in the present work—using a normobaric hypoxic tent (FiO₂ of 14.5%) for 18 h daily over 8 weeks—the LHTL model did not result in higher protein expression of HIF‐1α, PGC‐1α, and OXR1 in various tissues compared to training alone. Although no biomolecular changes related to oxidative metabolism were observed, this does not imply that the demands of the LHTL model are trivial. On the contrary, induced behavioral changes—such as reduced spontaneous physical activity and a diminished ability to complete training sessions—were more evident in mice exposed to the LHTL model than in those that trained and lived entirely in normoxia.

## AUTHOR CONTRIBUTIONS

In this study, Scariot P.P.M., Papoti M., Manchado‐Gobatto F.B., and Gobatto C.A. conceived the ideas, designed the experiments, and provide financial support. Scariot P.P.M, Polisel E.E.C. and Orsi J.B., contributed to data collection, performed data analysis, and interpreted the results. Scariot P.P.M, Papoti M., Hill D.W., Polisel E.E.C., Manchado‐Gobatto F.B. and Gobatto C.A. contributed to the discussion of data, and wrote the manuscript. All authors contributed to critical review of the manuscript, read and approved the final document.

## FUNDING INFORMATION

This study was financed in part by the Coordenação de Aperfeiçoamento de Pessoal de Nível Superior—Brasil (CAPES)—Finance Code 001. We would like to thank São Paulo Research Foundation—FAPESP (Grants no. 2015/00272‐6, 2015/01362‐9, 2017/10201‐4, 2019/05115‐7, 2023/02728‐3) and National Council for Scientific and Technological Development—CNPq (Process no. 307395/2013‐8, 302827/2015‐3, 444434/2024‐0) for financial support.

## CONFLICT OF INTEREST STATEMENT

The authors declare that they have no known competing financial interests or personal relationships that could have appeared to influence the work reported in this paper.

## ETHICS STATEMENT

All procedures and protocols were approved by an ethical review committee (CEUA‐UNICAMP, protocol number 5509–1/2020). All methods were carried out in accordance with relevant guidelines and regulations (Canadian Council on Animal Care guidelines and the Guide for the Care and Use of Laboratory Animals). The authors confirm that they have followed laws and standards for the protection of animals used for scientific purposes.

## Data Availability

Data will be made available on request.
